# Prediction of Soil Available Boron Content in Visible-Near-Infrared Hyperspectral Based on Different Preprocessing Transformations and Characteristic Wavelengths Modeling

**DOI:** 10.1155/2022/9748257

**Published:** 2022-08-11

**Authors:** Juanjuan Zhu, Xiu Jin, Shaowen Li, Yalu Han, Wenrui Zheng

**Affiliations:** ^1^Anhui Provincial Key Laboratory of Smart Agricultural Technology and Equipment, Anhui Agricultural University, Hefei 230036, Anhui, China; ^2^School of Information and Computer Science, Anhui Agricultural University, Hefei 230036, Anhui, China

## Abstract

The trace element boron (Boron, B) is an important factor in crops' development, pollination, and fertilization. Available boron (AB) in soil is the main source of boron nutrient absorption for crops. Rapid detection of AB is of great significance for crop nutrition diagnosis, soil testing and fertilization, precision agriculture development, scientific production management, and guarantee of stable yield and high quality. In this study, we propose a new method to predict soil available boron content using handheld nonimaging hyperspectroscopy in the visible-near-infrared range (350–1655 nm). As boron content is one of the fewest soil chemical elements, a rapid and accurate method has yet to be developed to detect and quantify the soil available boron. Visible-near-infrared ray (VIS-NIR) spectroscopy is widely utilized in the detection and quantification of soil available nutrients. There is, however, scant research on the detection of soil boron based on NIR data, and the performance of current regression model is still far from satisfactory. Our soil samples were collected from southern Anhui, China, with their NIR spectroscopy examined and the NIR data pretreated by 29 transformations and modeled with 10 regression algorithms. Of all the tested methods, SVM_RBF, BPNN, and PLS_RBF algorithms demonstrated the best performance and gave 0.80∼0.82 coefficient of determination value. At the same time, Random Forest algorithm (RFA), Successive Projection Algorithm (SPA), and Variable Importance in Projection (VIP) were used to extract the spectral characteristic wavelength data of soil available boron, and then the characteristic wavelength data were modeled with three regression algorithms: SVM_RBF, PLS_RBF, and BPNN. A comparative analysis of the prediction performance (*R*^2^, RPD, RMSE, and RPIQ) of the models established at the full band showed that the RFA-MSC/BPNN model achieved the best performance. Compared with the best full-wavelength model DT/SVM_RBF, the test set achieved a 3.06% increase in *R*^2^, a 7.12% drop in RMSE, a 7.71% gain in RPD, and a 7.78% increase in RPIQ. Our work sheds lights on how to achieve rapid quantification of the soil available boron concentration.

## 1. Introduction

As an essential trace element, boron plays a pivotal role in flowering, fertilization, yield boosting, and quality of crop produce [1]. In coarse-textured sandy calcareous soils, boron can serve as one of the key limiting micronutrients. Boron deficiency can be a major constraint on crop production [2] and is reported in >80 countries for at least 132 crops over the past 70 years [3]. Boron deficiency is a global phenomenon and is recognized as the second most essential micronutrient constraint on crops [4]. Researches demonstrated that 21% of the studied soils across 14 countries were boron deficient [5]. As boron content is usually the least among all the chemical elements in soil, a rapid and accurate detection of the soil available boron carries special weight. However, currently soil available boron detection methods (such as curcumin method and azomethine-H method) mainly depend on chemical analysis and thus are prone to low detection efficiency, high cost, sample damage, and potential environmental pollution.

Hyperspectroscopy mainly uses the characteristics of electromagnetic energy to analyze soil properties. Visible-near-infrared light is a kind of electromagnetic wave, and near-infrared spectroscopy is generated by vibrational and rotational energy level transitions in the internal movement of molecules. Present in boric acid molecules and boric acid ions, the soil available boron is mainly water-soluble and adsorbable by organic matters, which have unique spectral characteristics. Visible-near-infrared ray (VIS-NIR) spectroscopy can provide a nondestructive, real-time, rapid method to detect the physical and chemical properties in crops and soil [6]. VIS-NIR, therefore, is widely utilized in agriculture in monitoring the organic compounds and mineral nutrients.

VIS-NIR spectroscopy of soil nutrient elements mainly focuses on organic matter (OM), nitrogen (N), carbon (C), and water, and only a few studies have focused on the quantitative prediction of soil available boron. Mouazen et al. performed partial least square linear regression (PLSR), principal component regression (PCR), and back-propagation neural network (BPNN) comparative analysis on the accuracy of VNIR spectroscopy (350–2500 nm) in measuring soil properties, using 168 soil samples collected in Belgium and France for organic carbon (OC), phosphorus (P), potassium (K), sodium (Na), and magnesium (Mg). It was found that the prediction models of various attributes established by using the latent variables of PLS combined with BPNN were better than the PLSR and PCR models, and the PCR effect was the worst. Among them, the model accuracy of OC and Mg was higher, and the *R*^2^ values of the prediction results were 0.84 and 0.82, respectively; the RPD value was both 2.54 while the K, Na, and P models have *R*^2^ = 0.68–0.74 and RPD = 1.77–1.94; and the prediction effect is average [7]. Tarin et al. compared partial least squares discriminant analysis (PLS-DA), random forest (RFA), SG Simple Smoothing (SGS), SG Smoothed First Derivative (SG1D), SG Smoothed Second Derivative (SG2D), Mean Centering (MC), Standard Normal Transformation (SNV), Multiple Scatter Correction (MSC), Generalized Least Squares Weighting (GLSW), SG1D + SNV, and MSC + SG2D + MC. The soil spectral modeling performance of SGS + AS + GLSW with 10 different pretreatment transformations was evaluated for SOM, pH, NH_4_^+^, NO_3_^+^, and other soil properties in the Negev Desert of Israel. Results show that the GLSW-based model has relatively good classification accuracy, and both PLS-DA and RF are suitable for spectral modeling analysis [8]. Tahmasbian et al. utilized laboratory-based hyperspectral image (400–1000 nm) analysis to predict soil C, N, and their isotopic compositions; the PLSR models gave coefficient of determination (*R*^2^) > 0.8 for all tested compositions [9]. Tamburini group examined the eﬀects of moisture and particle size on quantitative determination of total organic carbon (TOC) in soils by near-infrared spectroscopy and discovered that standard normal variate (SNV) and second derivatives combined with the PLSR regression algorithm gave the best prediction [10]. Padarian et al. used a deep convolutional neural network (CNN) to establish NIR calibration models for OC, TN, cation exchange capacity (CEC), pH, clay, and sand content in soil and found that CNN had higher model accuracy compared with traditional models [11]. Qi et al. used VNIR spectra (350–2500 nm) data based on regularized linear multitask learning (LMTL) algorithm to model and predict available N, P and K, pH, water content (WC), OM, and electrical conductivity (EC). The performance of LMTL model was compared with the commonly used single-task algorithm model index based on PLSR, which shows that LMTL can further improve the generalization ability of regression model to predict soil properties [12]. Jin et al. utilized VIS-NIR spectroscopy for prediction of soil available K content and uncovered that the boosting algorithms (GBRT and AdaBoost) demonstrated the best *R*^2^ [13].

Though NIR has attracted enormous attention and has been studied intensively in soil disciplines over the past decades [14], the accuracy and universality of the VIS-NIR model to predict soil available minerals is still not satisfactory, especially for trace element content. Currently, Malmir et al. reported the utilization of hyperspectral imaging (400–1000 nm) technique to analyze the boron content in sieved and ground air-dried soils, modeled by the PLSR algorithm, and reached *R*^2^ of 0.62 and 0.53 in sieved and ground soils, respectively [15]. Airborne HSI and laboratory mid-infrared spectroscopy (2500–25,000 nm) generated *R*^2^ of 0.17∼0.30 for soil boron prediction [16, 17]. In contrast, the best model for As prediction can be achieved with 5 latent variables in PLS models and yielded Pearson's coefficient, RMSE, RPD, and SEP of 0.94, 69.65, 2.9, and 66.99, respectively [18].

In this study, a total of 188 yellow loam samples were collected from Anhui province, China. The nonimaging VIS-NIR spectrum was examined by indoor analysis, and the boron content was determined by chemical analysis. The spectrum data were transformed by 29 preprocessing methods, including detrend correction and Savitzky–Golay (SG) convolution smoothing and further modeled by 10 regression algorithms, such as elastic net, ridge, and support vector machine (SVM). The original spectra were preprocessed by DT, MSC, and SG + SNV + DT, respectively. Then, three different variable selection algorithms (RFA, SPA, and VIP) [19–21] were used to select a small number of characteristic wavelengths, and SVM_RBF, BPNN, and PLS_RBF were combined to establish nine models for comparative analysis. The established models were evaluated for the prediction of soil available boron by *R*^2^, ratio of performance of deviation, ratio of performance to IQ, etc. for the model's accuracy, reliability, and stability. Our work is one of the first studies to predict soil available boron in the 200–1700 nm range based on nonimaging hyperspectroscopy. Our results provide a reference for remote sensing monitoring of soil and fertilizer micronutrient element information.

## 2. Materials and Methods

### 2.1. Soil Sample Collection

**Figure 1:**
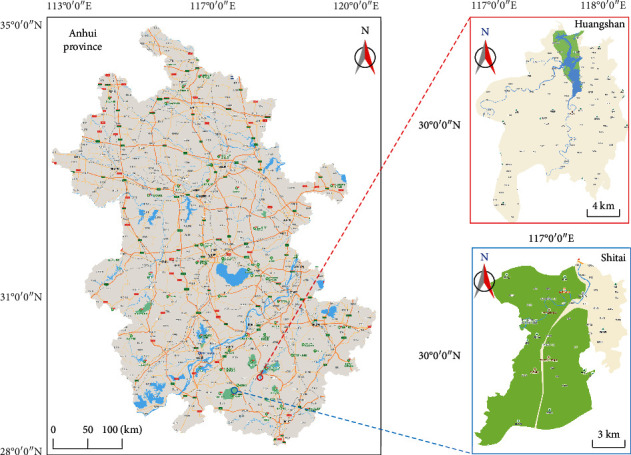
The sampling areas for soil collection in southern Anhui, China.

The experimental soil samples in this study were collected from rapeseed fields in southern Anhui. As a boron-preferred crop, rapeseed has high demand for boron. Because of its strong boron absorption capacity, rapeseed is highly sensitive to boron nutrition. A total of 188 yellow loam soil samples were collected from the typical mountainous region in southern Anhui, China (Figure 1). The geographical coordinates for sampling are 117°29′7″∼118°11′1″ E, 30°8′23″∼30°22′25″ N. Diagonal sampling method was utilized for sampling with its depth between 0 and 20 cm. After the removal of plant roots and grave debris, 1.5 kg pure soil samples were collected, numbered, air-dried, and ground. Samples > 2 mm in diameter were filtered. Each sample was passed through hyperspectral analysis and boron examination by VIS-NIR and azomethine-H acid colorimetric analysis.

**Figure 2:**
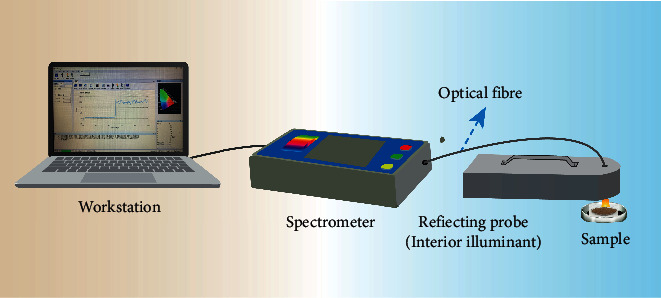
The laboratory visible-near-infrared spectroscopy acquisition system.

The VIS-NIR measurements were performed using a portable nonimaging spectrometer (Ocean Optics OFS-1700) with a spectral range 200–1700 nm (Figure 2). The spectral resolution is 2 nm for 200–950 nm and 5 nm for 950–1700 nm. The resampling interval is 1 nm. Measurements between 200 and 349 nm were filtered as noise.

Processed 2 mm soil powder particles were placed in a sample container which was covered with a black cloth to insulate it from stray light. For each soil sample, 3 sets were randomly selected for spectrum measurement, and the average spectra were utilized as the soil spectrum.

### 2.2. Pretreatment Transformation

A total of 29 methods were utilized for pretreatment transformation, including the sole application or combinations of detrend correction (DT), first derivative transformation (FD), second derivative transformation (SD), logarithmic transformation (LG), mean centering (MC), multivariate scattering correction (MSC), standard normal variable transformation (SNV), and Savitzky–Golay convolution smoothing (SG) (Table 1) [22, 23]. Among them, the SG treatment is generally utilized to remove the edge band from the spectral curve, which significantly eliminates the influence of high-frequency noise, enhances the signal-to-noise ratio, and maximally retains the peak characteristics of the original spectral signal. Even though FD and SD are effective in eliminating the linear baseline effect, the noise will be amplified after treatment. SNV is applied to calibrate the influence of soil particle size and surface scattering [24], while MC and DT reduce the spectral offset. Therefore, various treatments, when combined together, may integrate their strengths and eliminate their weaknesses.

### 2.3. Regression Algorithms

Totally, 10 algorithms were utilized for regression. As a common multiple linear regression algorithm [25], partial least squares (PLS) has been widely used in data analysis to predict soil properties using spectra. Support vector regression (SVR) is a popular algorithm in the machine learning field [26]. Different kernel functions, including linear, polynomial, sigmoid, and radial basis functions (RBF), are employed to map the inputs to a high-dimensional feature space.

**Figure 3:**
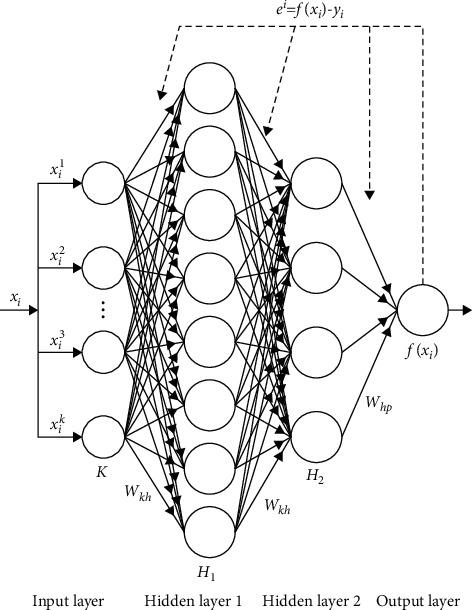
BPNN model architecture.

BPNN is a one-way multilayer perceptual feed-forward neural network [27], and its powerful learning ability has been widely used in soil spectral regression modeling analysis [28, 29]. In this study, a four-layer BPNN model is selected, including the input layer, the middle two hidden layers, and the output layer. The modeling structure of sample *i* is shown in Figure 3.

A BP neural network regression model of soil available boron based on the whole band was constructed by using 1306 wavelengths extracted from hyperspectral data in the effective band range of 350–1655 nm as the input of the model. The number of nodes in the input layer and the output layer was set to 16 and 1, respectively. The two hidden layers in the middle were set to 8 and 4 nodes, respectively. At the same time, tan-sigmoid is selected as the transfer function of the hidden layer, and pure-linear is selected as the transfer function of the output layer. In order to minimize the overfitting phenomenon, the Bayesian-regularized back-propagation algorithm (trainbr) was used for model calibration and training. The tuning parameters set the loss function to be Mean Squared Error, the initial learning rate to be 0.01, the learning rate to be 0.1, the momentum to be 0.9, and the maximum number of iterations to be 150; the optimizer is SGD, and SGD is random descent.  Table 2 shows the setting of network structure parameters.

Ridge regression estimates the coefficients of multiple-regression models when linear regression models have highly correlated independent variables by creating a ridge regression estimator, which provides a more accurate ridge parameter approximation.

Lasso regression performs both variable selection and regularization to enhance the model's prediction accuracy and interpretability. The lasso procedure encourages simple, sparse models with fewer parameters, and it is well-suited for models with high multicollinearity levels.

The elastic net is a regularized regression method that linearly integrates the penalties of the lasso and ridge methods to effectively shrink coefficients (such as in ridge regression) and set some coefficients to zero (such as in lasso).

### 2.4. Evaluation Metrics

The coefficient of determination (*R*^2^), the root mean square error (RMSE), and the ratio of performance of deviation (RPD) were adapted as prediction evaluation metrics in this study.

(1) $$\begin{array}{c} \text{RMSE}=\sqrt{\frac{\sum_{i=1}^{n}{\left(y_{i}-{\hat{y}}_{i}\right)}^{2}}{n}},\\ R^{2}=1-\frac{\sum_{i=1}^{n}{\left({\hat{y}}_{i}-y_{i}\right)}^{2}}{\sum_{i-1}^{n}{\left(y_{i}-\bar{y}\right)}^{2}},\\ \text{RPD}=\frac{S.D}{\text{RMSE}}=\sqrt{\frac{n\sum_{i=1}^{n}{\left(y_{i}-\bar{y}\right)}^{2}}{\left(n-1\right)\sum_{i=1}^{n}{\left(y_{i}-{\hat{y}}_{i}\right)}^{2}}}. \end{array}$$

In the formula, *n* is the number of predicted samples, *y*_*i*_ is the actual chemical measurement value of the *i* th sample, ${\hat{y}}_{i}$ is the predicted value of the *i* th sample, and $\bar{y}$ is the average value of *y*_*i*_.

S.D is the standard deviation. The models were categorized into different levels based on different RPD values as shown in Table 3.

Since the soil's physical properties and chemical contents usually demonstrate a biased normal distribution, the ratio of its performance to IQ (RPIQ) serves as a better indicator than RPD. RPIQ is the ratio of IQ to RMSE, where IQ is the difference between the third quartile Q3 (75% of samples) and the first quartile Q1 (25% of samples). The larger the value of RPIQ is, the better the performance of the model demonstrates. Nawar and Mouazen accessed the model quality based on RPIQ values [30]: excellent model (RPIQ ≥ 2.5), very good model (2.5 > RPIQ ≥ 2.0), better model (2.0 > RPIQ ≥ 1.7), a reasonable model (1.7 > RPIQ ≥ 1.4) and a very poor model (RPIQ < 1.4).

(2) $$\begin{array}{c} IQ=Q3-Q1,\\ \text{RPIQ}=\frac{IQ}{\text{RMSE}}\\ =\frac{Q3-Q1}{\sqrt{1/n\sum_{i=1}^{n}{\left(y_{i}-{\hat{y}}_{i}\right)}^{2}}}. \end{array}$$

In summary, this manuscript compares the *R*^2^, RMSE, RPD, and RPIQ for regression model comparison.

## 3. Results and Discussion

### 3.1. Soil Sample Statistics

By means of the Kennard-Stone method, these 188 soil samples were split into a training set and a testing set with a ratio of 7 : 3, namely a training set of 131 samples and a testing set of 57 samples. As the statistical metrics demonstrated in Table 4, both sets exhibited different distribution patterns in soil available boron content.

**Figure 4:**
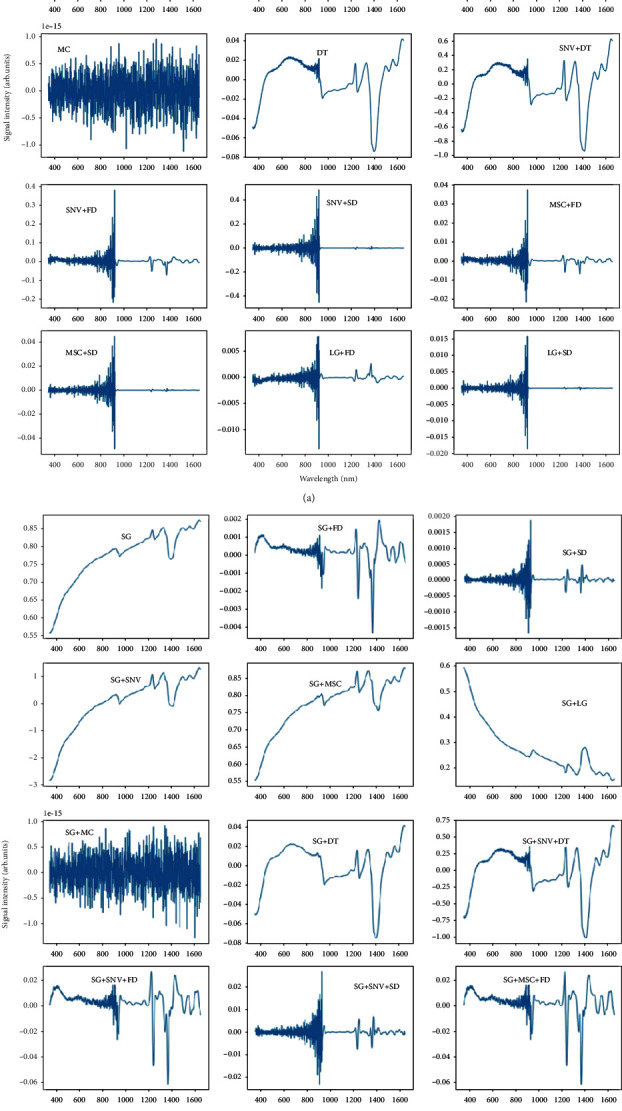
Average spectroscopy after various pretreatment transformations. (a) Average spectrum without Savitzky–Golay(SG) treatment; (b) average spectrum with SG method.

Pretreatment is an essential step in accurate VIS-NIR spectrum analysis. Various pretreatment methods were employed to filter noise and reduce complexity. Reflection spectra with diverse pretreatments are revealed in Figure 4. SG method can reduce spectrum noise and smooth the curve, and therefore, it is always used in combination with other pretreatment methods (Figure 4(b)). Except for the scattering correction methods, SNV and MSC, the rest of the methods all significantly modified the pattern of the spectral curve. FD, SD, and LG almost reshaped the curve thoroughly.

### 3.2. Performance Evaluation for Different Regression Models

**Figure 5:**
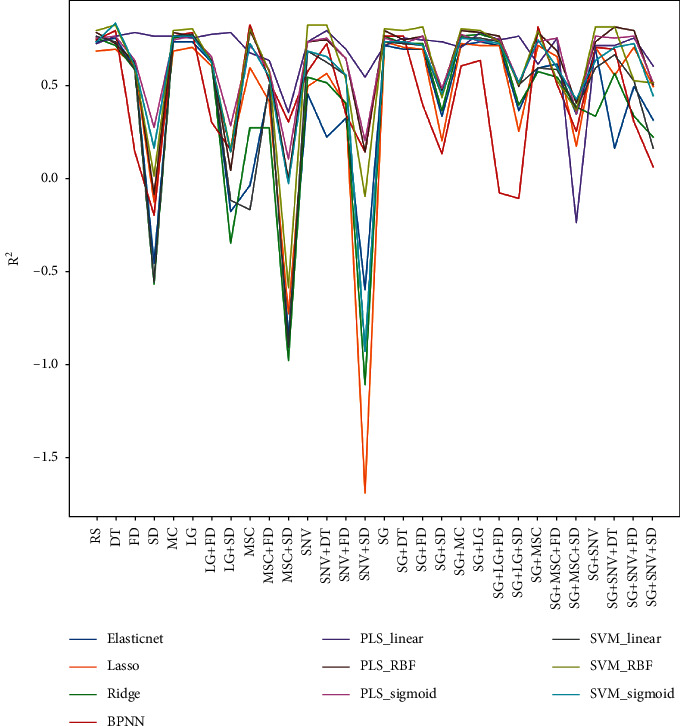
The accuracy of regression models with test data set by all pretreatment transformations.

**Figure 6:**
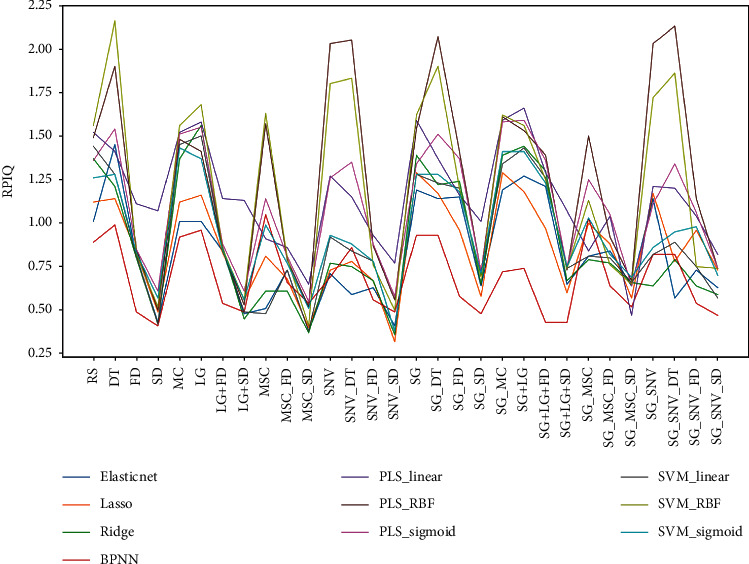
Ratio of performance to interquartile distance (RPIQ) values of regression models with different pretreatment transformations.

The combination of pretreatment transformation and regression algorithms generated a total of 300 models for the VIS-NIR spectrum.  Figure 5 exhibits the *R*^2^ values of each model for the test sets. The SVM method utilizing the RBF kernel and the PLS model with the RBF kernel demonstrated the highest *R*^2^ values in the prediction of test data no matter which pretreatment transformation was applied. Whatever regression model was employed, the pretreatments by SD, MSC + SD, or SNV + SD always generated the worst *R*^2^, especially for SNV + SD. The RPD levels and RPIQ of models are exhibited in Table 5 and Figure 6. Consistent with the *R*^2^ result, SVM with RBF kernel and PLS with RBF generated the most A-level results. The elastic net and lasso models did not compare favorably with other models in performance. Since the soil available boron content showed a biased normal distribution, the RPIQ is employed for evaluations in Figure 6. The SVM with RBF kernel dominated the best performance in almost every pretreatment group. The highest RPIQ value (2.16) appeared in the DT group with SVM_RBF model.

Table 5 demonstrates the Level A RPD level of each model to determine the influence of pretreatments. Level A indicated the highest stability for a model while Level C suggested the lowest stability. Without any pretreatment, some RS data sets can also reach A level (Supplementary Table). The elastic net, lasso, and SVM_Sigmoid models for RS data rendered Level C while ridge, SVM_Linear, and SVM_RBF models increased its level to B. After being pretreated by DT, LG, SNV, MSC, SNV + DT, SG + DT, or SG + SNV + DT, and further regressed by SVM_RBF, the VIS-NIR data could generate Level A model (Supplementary Table). This indicated that DT or SNV was more preferable to other pretreatment transformations.

**Figure 7:**
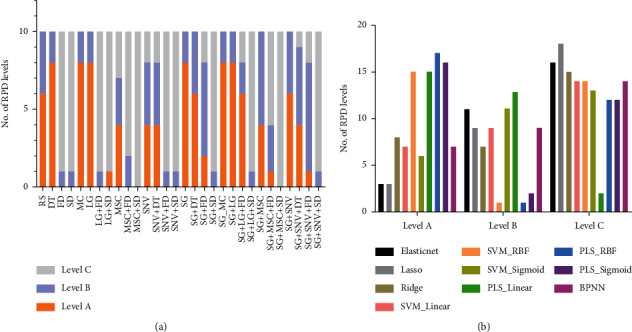
The statistics of RPD levels under different treatments.

Figure 7(a) demonstrates the statistics of RPD levels based on the pretreatment types. Even though pretreatment transformations were expected to reduce the noise and increase the accuracy, several transformations generated worse results than the original RS spectrum, especially for FD and SD. Most transformations containing FD and SD led to all Cs, which strongly indicated that these two transformations cannot be used to predict boron based on VIS-NIR data. DT and LG methods improved the overall performance to better levels compared to the original RS data. The performance of MSC and SNV improved in some models but declined in others. No observable improvement was detected for the SG treatment, even though it was the typical pretreatment utilized in NIR data analysis.

Figure 7(b) shows the statistical result of RPD levels for different based on regression methods. PLS models generated the most A-level results, which suggested its stability in prediction.

### 3.3. The Favorable Models for VIS-NIR Prediction of Boron

Different regression algorithms were combined with different pretreatments to generate the best model for each regression algorithm. Elastic net and SVM_RBF were required to combine with DT pretreatment to render the best model while ridge, SVM_Linear, and SVM_Sigmoid were the best partners for LG. SG-transformed methods were preferential for PLS. SG was the best choice for lasso regression (Table 6). All of these combinations resulted in a *R*^2^ ≥ 0.72, and SVM_RBF generated both the highest *R*^2^ (0.82) and the best RPD level (Level A). Therefore, dissimilar regression algorithms corresponded to diverse pretreatments to achieve the optimal performance, and DT + SVM_RBF rendered the best performance among all the tested models in this study (Table 6). Consistent with the *R*^2^ and RPD-level result, the RPIQ values of SVM_RBF were the highest among these models (Table 6). In summary, the SVM_RBF algorithm was determined to exhibit the best performance in predicting the soil available boron content by VIS-NIR.

### 3.4. Spectral Feature Extraction of Soil Available Boron

RFA is an ensemble machine learning approach, which uses its variable importance measure as a feature selection tool for high-dimensional data sets to sort the feature data, search one by one in a sequential backward way, and eliminate the least influential features from the feature set in turn by recursive iteration. As the number of variables in the characteristic wavelength data set increases, the classification accuracy keeps growing accordingly until the optimal characteristic variables are selected. RFA has a very flexible, powerful, efficient, and practical classification feature ability, and it is also robust to identify some data with missing outliers and noise data, and its learning and iterative optimization speed is fast. In recent years, RFA has been mainly used to solve various problems such as classification, prediction, feature selection, outlier detection, and recognition [19, 31].

As a forward selection variable method that minimizes vector space collinearity, SPA selects wavelengths to reduce information redundancy and solve the linear problem. According to the method, variable groups with redundant information can be sufficiently and thoroughly eliminated from a large amount of spectral information, and original spectral data are replaced by residual spectral information so as to reduce data dimensionality and the number of data variables. The above method has been widely used in spectral analysis. Using the Monte Carlo sampling method, a certain proportion of the wavelength data was extracted for PLS modeling, and the absolute values of the regression coefficients were compared. The spectral wavelength variables with small absolute values were eliminated for their small weights, while the spectral wavelength variables with large absolute values were retained because of their large weights. After the *i*th Monte Carlo sampling, the minimum was cross-validated with the root mean square error to determine the optimal modeling wavelength [32]. In this study, the Monte Carlo sampling was run 100 times, and the number of characteristic wavelengths was determined by 10-fold cross-validation.

VIP technology [21] is a variable screening method based on partial least squares regression, and its value can be used to identify some important wavelengths in the model. Its specific calculation and formula are as follows:

(3) $$\begin{array}{c} V_{k}\left(a\right)=p\sum_{a}w_{ak}^{2}\left(\frac{SSY_{a}}{SSY_{t}}\right), \end{array}$$

where *V*_*k*_(*a*) is the score of the projection importance of the *k*-th independent variable under the condition of using *a* latent variables for modeling, *p* is the number of independent variables, *w*_*ak*_ is the corresponding weight coefficient, *SSY*_*a*_ is the explanatory power of using *a* latent variables to the dependent variable *y*, and *SSY*_*t*_ is the explanatory power of using all latent variables to *y*.

The VIP value represents the importance of the independent variable to the model fitting. It is generally believed that when all VIP values are equal to 1, the prediction effect of each variable on *y* is the same. When the VIP value is greater than 1, the independent variable has a very important indication effect on the prediction of *y*, that is, the characteristic wavelength; when the VIP value is less than 1, it means that the contribution of the independent variable to the prediction of *y* is small. The researcher Word [33] believes that the contribution of the independent variable to *y* can be ignored when the VIP value is less than 0.8. VIP analysis is widely used in independent variable screening in various fields, and scholars such as Paz-Kagan et al. [34] and Rossel et al. [35] also use VIP values to analyze the corresponding relationship between different spectral bands and the detected objects. The larger the VIP value is, the stronger the importance of the wavelength in the prediction model is, and the smaller the number of characteristic wavelengths is.

In order to compare with the three better methods in the results in Section 3.3, on the basis of DT, MSC, and SG + SNV + DT preprocessing of the original spectrum, three different variable selection algorithms (RFA, SPA, VIP) were selected to screen out a small number of characteristic wavelengths. The prediction results are shown in Tables 7–9 by combining the nine models established by the three algorithms of SVM_RBF, BPNN, and PLS_RBF.

**Figure 8:**
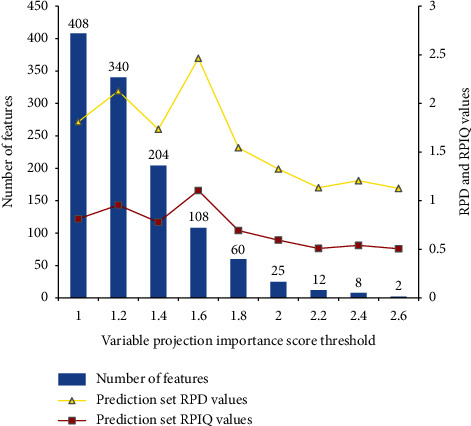
Effects of different thresholds of variable importance in projection on models' performance.

In MSC/BPNN modeling, the characteristic wavelengths selected by the VIP threshold between 1.0 and 1.6 have strong collinearity; when the threshold exceeds 1.6 (corresponding to 108 wavelengths), the learning ability of the model begins to deteriorate significantly. Therefore, the threshold value of 1.6 was selected as the VIP value, and 108 characteristic wavelengths were used as the input data of BPNN to establish the regression model of soil AB.  Figure 8 shows the results of the impact of different variable projection importance score thresholds on the accuracy of the VIP-MSC/BPNN model.

**Figure 9:**
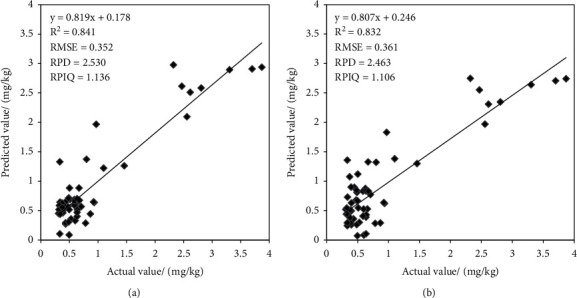
Comparison of predicted values and actual values of VIP-MSC/BPNN and RFA-MSC/BPNN models.

Based on the RFA-MSC/BPNN model, the predicted soil AB content in the test set was compared with the actual detection value, and the results are shown in Figure 9(a). Figure 9(b) shows the results of the comparison between the predicted value of the VIP-MSC/BPNN model and the actual detection value. It can be seen that there is a good correlation between them. The AB content was mostly concentrated in the lower value area, which is consistent with the distribution patterns of micronutrient content.

## 4. Discussion

Based on the VIS-NIR spectroscopy of soil samples collected from China's Anhui province, this study combined 29 pretreatment transformations, the original RS data, with 10 regression algorithms to generate 300 models for the prediction of soil available boron contents. Among all the generated models, the SVM_RBF model with DT pretreatment, PLS_RBF model with SG_SNV_DT transformation, and the BPNN model with MSC pretreatment significantly outperformed other models and gave *R*^2^ value of 0.80 to 0.82 and RPD Level A (Table 6). SVM is widely used for the calibration of VIS-NIR spectra [24, 36], and the nonlinear RBF kernel is a Gaussian kernel. Since the number of samples in our study is much smaller than that of features, the number of frequencies, the Gaussian kernel here played the role of dimensionality reduction. The performance of the PLS_RBF model is literally similar to that of SVM_RBF when *R*^2^, RMSE, RPD, and RPIQ metrics are utilized for performance evaluation (Table 6). The two best models utilize the RBF model, which suggests that the Gaussian kernel is effective in predicting soil available mineral and also solidifies the necessity of dimensionality reduction for soil content prediction. The DT pretreatment method filters the tendency and reflects the true fluctuation and thus can eliminate the deceptive correlation. In addition, DT transformation usually follows SNV, and our results also demonstrate that SNV alone and SNV + DT pretreatments in the SVM_RBF models show Level A results (Table 5). Generally, the DT pretreatment seems to be able to improve the model performance when superimposed with other transformations, whatever the regression algorithm is employed. This result also suggests that SNV, when well-tuned with the regression algorithms, may generate an acceptable model for soil boron prediction.

Meanwhile, the PLS_Linear model generated the fewest Level C results (Figure7(b)), which was consistent with our previous results for soil K prediction [13]. Even though the RBF kernel (SVM or PLS) generated the most Level A models, they also generated a comparable number of Level C counterparts (Figure 7(b)). This indicated that the RBF kernel may be especially suitable for some, but not all, pretreatments. In contrast, the PLS_Linear model may be more applicable for all pretreatment transformations. PLS_Linear model, therefore, has stronger robustness.

Even though the pretreatment transformations were expected to smooth the curve, reduce noise, and improve model performance, not all pretreatments were effective in our study of soil available boron prediction. As a standard preparation of the soil spectral curves, SG is utilized in almost every NIR analysis. However, our results demonstrated that SG contributed little to model performance improvement. In some models, it even resulted in worse performance, such as DT + SG vs. DT alone in the elastic net models (Table 5). Additionally, the SD transformation caused severe performance reduction in almost every model, which strongly indicated that this method was inappropriate for the analysis of soil available chemical content prediction based on VIS-NIR.

**Figure 10:**
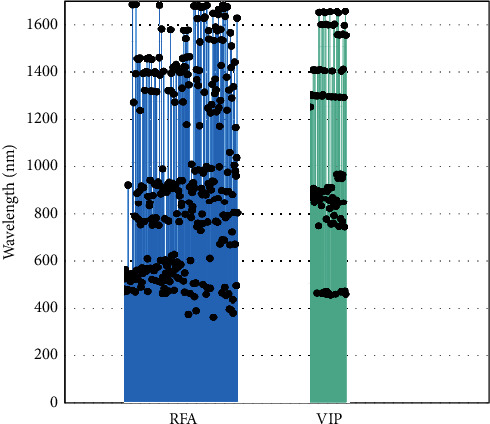
Distribution of characteristic wavelength points.

Based on the above results, it was found that 328 characteristic wavelengths extracted by the RFA algorithm can be used as the best characteristic wavelengths of soil AB content. The specific characteristic wavelengths and distribution points selected by RFA and VIP during MSC/BPNN modeling are arranged in order of importance of VIP values from large to small as shown in Table 10 and Figure 10. It is found that the AB characteristic wavelengths of soil are mainly distributed in 400–600 nm, 700–1000 nm, 1300–1400 nm, and 1500–1700 nm, including 455 nm, 538 nm, 858 nm, 905 nm, 1645 nm, and other important wavelengths. The distribution of characteristic wavelengths selected by VIP is relatively more concentrated and obvious, and the main characteristic wavelengths are distributed around 450 nm, 850 nm, 1300 nm, 1400 nm, 1600 nm, and 1650 nm. Some literature shows that the dissociation of boric acid at low concentration will be adsorbed by clay minerals, iron and aluminum oxides, and organic matters in combination with the OH group [37, 38]. However, the AB characteristic wavelength of the soil in this study is highly consistent with the absorption band of iron oxide and hydroxyl OH, with similar spectral peaks, which is consistent with the research results of Beyrouty et al. [39], indicating that the AB spectral characteristic response may be related to boron adsorption. In addition, Tahmasbian et al. [9, 40] have shown that the spectral regions of 400–410 nm, 515–575 nm, 660–665 nm, 875 nm, and 910–1000 nm are important wavelengths for predicting soil TN. The 940–1000 nm region is one of the most important regions for soil TC prediction. Shi et al. [41] found that the TN content showed a high correlation with the derivative spectra and that the important absorption wavelengths were near the visible 540 nm and near-infrared 1400, 1900, 2200, and 2300 nm regions. Yang and Li and Cozzolino and Morón [42, 43] found that 700–1000 nm is also an important band range for TC prediction. There is also an overlap between the important wavelengths of soil AB in the current study and those of TC and TN in previous studies, indicating that there is a high correlation between soil AB spectral analysis and soil TC and TN. The overlap of important spectral regions and the strong correlation between the successfully predicted elements indicate that the successful prediction of AB may also result from its high degree of correlation with the spectrally active compounds *C* and *N* in soil.

Currently, there is limited research on NIR-based boron content detection in soil. Relevant research is found in only a few groups' work. However, the accuracy was not high enough [15]. With *C* = 200000 and gamma = 1 as parameters, we improved the *R*^2^ value in our model to 0.82. By modeling with the selected characteristic wavelength, we further improved the *R*^2^ value to 0.84 in the model, which is much higher than that was used in the Malmir model. Since boron is one of the fewest elements in soil, predicting its available content and total content is quite a challenging task. Our research generates two models with high *R*^2^ and low RMSE, which lay the groundwork for rapid detection of soil boron.

## 5. Conclusions

Based on the VIS-NIR data of 188 soil samples collected from southern Anhui, China, 300 regression models were generated for soil available boron prediction by the assembly of 29 pretreatment methods, plus the original spectrum reflectance data set, and 10 regression algorithms. The most favorable models for soil boron content prediction were generated from the DT-pretreated spectrum data followed by the SVM algorithm with RBF kernel function, the MSC transformations followed by the BPNN, or the SG_SNV_DT transformations followed by PLS with RBF kernel. With the parameters of C 200000 and gamma 1 for the SVM_RBF model, [1, 4, 8, 16] for BPNN and n_component 14, gamma 0.05 for PLS_RBF, a high *R*^2^ value of 0.80–0.82, and RPD Level A were reached. SVM_RBF, BPNN, and PLS_RBF algorithms were considerably superior to other algorithms in our study, and SD pretreatment caused inferior performance in most cases. Even though SG transformation is generally employed in the NIR data analysis, no recognizable improvement was observed in the soil boron prediction models. Lasso and elastic net models are not suitable for the spectral prediction of soil AB.

In the study of hyperspectral prediction of soil available boron based on characteristic wavelength modeling, the original spectra were preprocessed by DT, MSC, and SG + SNV + DT. Three different variable selection algorithms (RFA, SPA, and VIP) were used to select a small number of characteristic wavelengths, and then, nine models were established by SVM_RBF, BPNN, and PLS_RBF. Results show that, RFA-MSC/BPNN (*N* = 328, *R*^2^  = 0.841, RMSE = 0.352, RPD = 2.530, and RPIQ = 1.136) and VIP-MSC/BPNN (*N* = 108, *R*^2^  = 0.832, RMSE = 0.361, RPD = 2.463, and RPIQ = 1.106), the prediction accuracy of the two models was further improved on the basis of the model accuracy constructed by the whole band, and the prediction accuracy grade reached A level, which could be used to predict the AB content of the soil. RFA-MSC/BPNN model generates the best effect, and compared with other modeling algorithms, the BPNN algorithm is better in the use of soil AB spectral feature extraction modeling method.

The study also shows that the successful prediction of AB may also be related to boron adsorption such as iron oxide and hydroxyl and has a high correlation with the spectral active compounds *C* and *N* in soil.

## Figures and Tables

**Table 1:** Pretreatment methods utilized for the visible-near-infrared ray spectroscopy of collected soil samples.

Pretreatment methods	Abbreviations
Reflection spectrum without pretreatment method	RS
Dislodge tendency	DT
First derivative	FD
Second derivative	SD
Mean center	MC
Logarithmic transformation	LG
First derivative with logarithmic transformation	LG + FD
Second derivative with logarithmic transformation	LG + SD
Multiplicative scatter correction	MSC
First derivative with multiplicative scatter correction	MSC + FD
Second derivative with multiplicative scatter correction	MSC + SD
Standard normal variate	SNV
Dislodge tendency with standard normal variate	SNV + DT
First derivative with standard normal variate	SNV + FD
Second derivative with standard normal variate	SNV + SD
Savitzky–Golay	SG
Dislodge tendency with Savitzky–Golay	SG + DT
First derivative with Savitzky–Golay	SG + FD
Second derivative with Savitzky–Golay	SG + SD
Mean center with Savitzky–Golay	SG + MC
Logarithmic transformation with Savitzky–Golay	SG + LG
First derivative with logarithmic transformation and Savitzky–Golay	SG + LG + FD
Second derivative with logarithmic transformation and Savitzky–Golay	SG + LG + SD
Multiplicative scatter correction with Savitzky–Golay	SG + MSC
First derivative with multiplicative scatter correction and Savitzky–Golay	SG + MSC + FD
Second derivative with multiplicative scatter correction and Savitzky–Golay	SG + MSC + SD
Standard normal variate with Savitzky–Golay	SG + SNV
Dislodge tendency with standard normal variate and Savitzky–Golay	SG + SNV + DT
First derivative with standard normal variate and Savitzky–Golay	SG + SNV + FD
Second derivative with standard normal variate and Savitzky–Golay	SG + SNV + SD

**Table 2:** BPNN modeling parameters.

Network layer	Number of nodes	Number of parameters
0	16	20912
1	8	136
2	4	36
3	1	5

**Table 3:** The categories of different models based on RPD values.

RPD	Level
RPD ≤ 1.4	C
1.4 < RPD ≤ 2.0	B
RPD > 2.0	A

**Table 4:** Soil available boron sample statistics.

Type	Number	Max (mg·kg^−1^)	Min (mg·kg^−1^)	Average (mg·kg^−1^)	Standard deviation
Total	188	3.91	0.24	0.87	0.86
Train	131	3.91	0.24	0.96	0.93
Test	57	3.65	0.28	0.68	0.66

**Table 5:** Level A RPD levels of regression models with various pretreatment transformations.

Pretreatment methods	Regression algorithms
DT	Elastic net, BPNN, SVM_Linear, SVM_RBF, SVM_Sigmoid, PLS_Linear, PLS_RBF, and PLS_Sigmoid
LG + SD	PLS_Linear
LG	Ridge, BPNN, SVM_Linear, SVM_RBF, SVM_Sigmoid, PLS_Linear, PLS_RBF, and PLS_Sigmoid
MC	Ridge, BPNN, SVM_Linear, SVM_RBF, SVM_Sigmoid, PLS_Linear, PLS_RBF, and PLS_Sigmoid
MSC	BPNN, SVM_RBF, PLS_RBF, PLS_Sigmoid
RS	Ridge, SVM_Linear, SVM_RBF, PLS_Linear, PLS_RBF, and PLS_Sigmoid
SNV	SVM_RBF, PLS_Linear, PLS_RBF, and PLS_Sigmoid
SNV_DT	SVM_RBF, PLS_Linear, PLS_RBF, and PLS_Sigmoid
SG + LG + FD	Ridge, SVM_RBF, SVM_Sigmoid, PLS_Linear, PLS_RBF, and PLS_Sigmoid
SG + LG	Elastic net, ridge, SVM_Linear, SVM_RBF, SVM_Sigmoid, PLS_Linear, PLS_RBF, and PLS_Sigmoid
SG	Lasso, ridge, BPNN, SVM_Linear, SVM_RBF, PLS_Linear, PLS_RBF, and PLS_Sigmoid
SG_DT	Ridge, BPNN, SVM_RBF, PLS_Linear, PLS_RBF, and PLS_Sigmoid
SG_FD	PLS_RBF and PLS_Sigmoid
SG_MC	Lasso, ridge, SVM_Linear, SVM_RBF, PLS_Linear, PLS_RBF, and PLS_Sigmoid
SG_MSC	BPNN, SVM_RBF, PLS_RBF, and PLS_Sigmoid
SG_MSC_FD	PLS_Linear
SG_SNV	Elastic net, lasso, SVM_RBF, PLS_Linear, PLS_RBF, and PLS_Sigmoid
SG_SNV_DT	SVM_RBF, PLS_Linear, PLS_RBF, and PLS_Sigmoid
SG_SNV_FD	PLS_RBF

**Table 6:** The performance and parameters of the best models.

Regression model	Pretreatment method	Test *R*^2^	Test RMSE	RPD level	RPIQ	Parameters
Elastic net	DT	0.75	0.09	A	1.45	Alpha = 2 *∗* 10^−5^, *L*1 = 0.01
Lasso	SG	0.72	0.12	A	1.30	Alpha = 0.0001
Ridge	LG	0.77	0.08	A	1.56	Alpha = 0.0005
**BPNN**	**MSC**	**0.81**	**0.37**	**A**	**1.55**	**[16, 8, 4, 1]**
SVM_Linear	LG	0.76	0.08	A	1.50	*n*_components = 3500
**SVM_RBF**	**DT**	**0.82**	**0.04**	**A**	**2.15**	**C** = **200000, gamma** = **1**
SVM_Sigmoid	LG	0.76	0.10	A	1.37	Gammas = 5 *∗* 10^−5^, *C* = 6200000, coef = 0
PLS_Linear	SG + LG	0.78	0.07	A	1.66	*n*_components = 14
**PLS_RBF**	**SG** + **SNV** + **DT**	**0.80**	**0.04**	**A**	**2.12**	**n_components** = **14, gamma** = **0.05**
PLS_Sigmoid	SG_MC	0.77	0.07	A	1.58	*n*_components = 15, gamma = 0.002, coef = 0

*Note.* Bold indicates that the prediction accuracy of the model is good.

**Table 7:** Result of DT/SVR_RBF models.

Variable selection method	No. of variables	Calibration sets				Test sets			
		*R* ^2^	RMSE	RPD	RPIQ	*R* ^2^	RMSE	RPD	RPIQ
Full wavelengths	1306	0.989	0.008	9.490	5.138	0.821	0.042	3.287	2.155
RFA	18	0.988	0.095	9.034	4.937	0.594	0.563	1.582	0.710
SPA	2	0.749	0.428	2.003	1.094	0.735	0.534	1.861	0.786
VIP	32	0.988	0.095	9.017	4.928	0.666	0.510	1.745	0.783

**Table 8:** Result of SG + SNV + DT/PLS_RBF models.

Variable selection method	No. of variables	Calibration sets				Test sets			
		*R* ^2^	RMSE	RPD	RPIQ	*R* ^2^	RMSE	RPD	RPIQ
Full wavelengths	1306	0.988	0.009	8.963	4.800	0.810	0.043	3.217	2.126
RFA	367	0.690	0.476	1.803	0.985	0.819	0.376	2.370	1.064
SPA	3	0.542	0.578	1.483	0.810	0.782	0.412	2.159	0.969
VIP	367	0.690	0.476	1.803	0.985	0.819	0.376	2.370	1.064

**Table 9:** Result of MSC/BPNN models.

Variable selection method	No. of variables	Calibration sets				Test sets			
		*R* ^2^	RMSE	RPD	RPIQ	*R* ^2^	RMSE	RPD	RPIQ
Full wavelengths	1306	0.764	0.415	2.066	1.129	0.816	0.379	2.349	1.054
**RFA**	**328**	**0.788**	**0.394**	**2.179**	**1.191**	**0.841**	**0.352**	**2.530**	**1.136**
SPA	24	0.733	0.441	1.944	1.063	0.736	0.453	1.964	0.882
**VIP**	**108**	**0.740**	**0.436**	**1.967**	**1.075**	**0.832**	**0.361**	**2.463**	**1.106**

*Note.* Bold indicates that the prediction accuracy of the model is good.

**Table 10:** Characteristic wavelengths selected by RFA and VIP in MSC/BPNN modeling.

Variable selection method	No. of variables	Characteristic wavelengths (nm)
RFA	328	538, 456, 466, 510, 464, 548, 535, 525, 513, 458, 460, 529, 465, 905, 463, 519, 518, 527, 520, 526, 498, 522, 530, 515, 528, 1654, 1653, 461, 521, 1248, 524, 523, 508, 769, 455, 1367, 1655, 545, 517, 871, 1429, 509, 758, 825, 1428, 879, 547, 1432, 1215, 734, 475, 900, 495, 555, 536, 532, 1369, 895, 539, 747, 549, 1298, 552, 546, 540, 1376, 551, 1430, 593, 457, 859, 858, 1371, 1433, 813, 828, 925, 554, 1295, 756, 1427, 507, 733, 760, 862, 1294, 736, 543, 752, 909, 1375, 1368, 502, 762, 560, 1292, 904, 906, 558, 917, 499, 732, 1434, 1651, 1361, 902, 586, 869, 494, 1553, 504, 449, 972, 516, 888, 1378, 583, 469, 759, 503, 588, 533, 450, 896, 876, 563, 512, 908, 1297, 749, 459, 916, 541, 497, 913, 1550, 1296, 1370, 605, 584, 891, 531, 912, 1394, 1282, 462, 610, 1249, 570, 550, 907, 1406, 1396, 779, 578, 514, 557, 1379, 882, 511, 746, 860, 1372, 924, 569, 1374, 814, 829, 1307, 923, 1431, 1250, 500, 1548, 1397, 454, 534, 777, 1395, 1513, 1435, 1156, 1547, 1363, 1365, 1549, 362, 1321, 830, 1437, 897, 448, 585, 901, 764, 992, 792, 875, 809, 914, 915, 490, 1648, 435, 893, 964, 1647, 492, 377, 1384, 1343, 726, 1596, 741, 1318, 1649, 968, 1381, 1151, 1499, 967, 710, 1642, 892, 870, 778, 865, 739, 486, 955, 1645, 1597, 1290, 1602, 872, 971, 864, 445, 982, 1650, 472, 745, 1225, 911, 1546, 1511, 833, 899, 594, 470, 1205, 885, 974, 1239, 1323, 921, 918, 1617, 350, 919, 1505, 844, 1284, 1345, 1561, 748, 1300, 1209, 1552, 1545, 744, 849, 1612, 1634, 1226, 981, 1149, 654, 1256, 1551, 1402, 1508, 883, 451, 672, 1506, 1652, 774, 1637, 670, 831, 474, 1605, 763, 440, 878, 1353, 1216, 1644, 958, 773, 880, 447, 1299, 384, 1041, 650, 1537, 381, 1393, 1482, 1266, 704, 866, 423, 367, 1313, 785, 988, 1415, 653, 963, 1144, 943, 481, 1019, 1598, and 783.

VIP	108	872, 1297, 1248, 1296, 860, 1298, 891, 875, 1295, 1403, 905, 859, 1404, 839, 861, 1402, 862, 1294, 883, 864, 847, 458, 1293, 1401, 871, 895, 741, 1644, 1645, 888, 1405, 853, 863, 1594, 825, 457, 1292, 1593, 1299, 460, 893, 1646, 461, 462, 1400, 1643, 455, 858, 1595, 769, 451, 464, 892, 909, 459, 1592, 1291, 456, 848, 813, 1647, 450, 449, 749, 908, 1290, 1399, 866, 844, 1591, 752, 833, 784, 1596, 851, 1289, 454, 822, 857, 964, 1648, 1551, 963, 947, 1642, 1552, 738, 1550, 1288, 840, 965, 771, 463, 1398, 465, 946, 760, 962, 1553, 1406, 843, 1287, 1590, 736, 466, 1649, 453, and 1549.

## Data Availability

The [DATA TYPE] data used to support the findings of this study are available from the corresponding author upon request.

## Supplementary Materials

Supplementary Materials Supplementary Table 1: RPD levels of regression models with various pretreatment transformations.
