# Supplementary material for: Prediction of Soil Available Boron Content in Visible-Near-Infrared Hyperspectral Based on Different Preprocessing Transformations and Characteristic Wavelengths Modeling
Source: Comput Intell Neurosci. 2022 Aug 11;2022:9748257. doi: 10.1155/2022/9748257 (PMC9388244; doi:10.1155/2022/9748257)
Supplement: Supplementary Materials — Supplementary Table 1: RPD levels of regression models with various pretreatment transformations. [file 9748257.f1.docx]

**Supplementary Table 1** RPD levels of regression models with various pretreatment transformations

| Pretreatment methods | Elasticnet | Lasso | Ridge | BPNN | SVM_Linear | SVM_RBF | SVM_Sigmoid | PLS_Linear | PLS_RBF | PLS_Sigmoid |
| --- | --- | --- | --- | --- | --- | --- | --- | --- | --- | --- |
| RS | B | B | A | B | A | A | B | A | A | A |
| DT | A | B | B | A | A | A | A | A | A | A |
| FD | C | C | C | C | C | C | C | B | C | C |
| SD | C | C | C | C | C | C | C | B | C | C |
| MC | B | B | A | A | A | A | A | A | A | A |
| LG | B | B | A | A | A | A | A | A | A | A |
| LG+FD | C | C | C | C | C | C | C | B | C | C |
| LG+SD | C | C | C | C | C | C | C | A | C | C |
| MSC | C | B | C | A | C | A | B | B | A | A |
| MSC+FD | C | C | C | B | C | C | C | B | C | C |
| MSC+SD | C | C | C | C | C | C | C | C | C | C |
| SNV | C | C | B | B | B | A | B | A | A | A |
| SNV+DT | C | C | B | B | B | A | B | A | A | A |
| SNV+FD | C | C | C | C | C | C | C | B | C | C |
| SNV+SD | C | C | C | C | C | C | C | B | C | C |
| SG | B | A | A | A | A | A | B | A | A | A |
| SG+DT | B | B | A | A | B | A | B | A | A | A |
| SG+FD | B | C | B | C | B | C | B | B | A | A |
| SG+SD | C | C | C | C | C | C | C | B | C | C |
| SG+MC | B | A | A | B | A | A | A | A | A | A |
| SG+LG | A | B | A | B | A | A | A | A | A | A |
| SG+LG+FD | B | C | A | C | B | A | A | A | A | A |
| SG+LG+SD | C | C | C | C | C | C | C | B | C | C |
| SG+MSC | B | B | B | A | B | A | B | B | A | A |
| SG+MSC+FD | C | C | C | B | C | C | C | A | B | B |
| SG+MSC+SD | C | C | C | C | C | C | C | C | C | C |
| SG+SNV | A | A | B | B | B | A | B | A | A | A |
| SG+SNV+DT | B | C | B | B | B | A | B | A | A | A |
| SG+SNV+FD | B | B | C | C | B | B | B | B | A | B |
| SG+SNV+SD | C | C | C | C | C | C | C | B | C | C |
